# Nuclear FGFR2 Interacts with the MLL-AF4 Oncogenic Chimera and Positively Regulates *HOXA9* Gene Expression in t(4;11) Leukemia Cells

**DOI:** 10.3390/ijms22094623

**Published:** 2021-04-28

**Authors:** Tiziana Fioretti, Armando Cevenini, Mariateresa Zanobio, Maddalena Raia, Daniela Sarnataro, Fabio Cattaneo, Rosario Ammendola, Gabriella Esposito

**Affiliations:** 1 CEINGE Advanced Biotechnologies s.c. a r.l., via G. Salvatore, 486, 80145 Naples, Italy; fioretti@ceinge.unina.it (T.F.); armando.cevenini@unina.it (A.C.); raia@ceinge.unina.it (M.R.); daniela.sarnataro@unina.it (D.S.); 2 Department of Molecular Medicine and Medical Biotechnologies, University of Naples Federico II, Via S. Pansini, 5, 80131 Naples, Italy; mt.zanobio@gmail.com (M.Z.); fabio.cattaneo@unina.it (F.C.); rosario.ammendola@unina.it (R.A.)

**Keywords:** AF4, cell culture, FGFR2, *HOXA9*, MLL-AF4, nucleus, target therapy, t(4;11) leukemia

## Abstract

The chromosomal translocation t(4;11) marks an infant acute lymphoblastic leukemia associated with dismal prognosis. This rearrangement leads to the synthesis of the MLL-AF4 chimera, which exerts its oncogenic activity by upregulating transcription of genes involved in hematopoietic differentiation. Crucial for chimera’s aberrant activity is the recruitment of the AF4/ENL/P-TEFb protein complex. Interestingly, a molecular interactor of AF4 is fibroblast growth factor receptor 2 (FGFR2). We herein analyze the role of FGFR2 in the context of leukemia using t(4;11) leukemia cell lines. We revealed the interaction between MLL-AF4 and FGFR2 by immunoprecipitation, western blot, and immunofluorescence experiments; we also tested the effects of FGFR2 knockdown, FGFR2 inhibition, and FGFR2 stimulation on the expression of the main MLL-AF4 target genes, i.e., *HOXA9* and *MEIS1*. Our results show that FGFR2 and MLL-AF4 interact in the nucleus of leukemia cells and that FGFR2 knockdown, which is associated with decreased expression of *HOXA9* and *MEIS1*, impairs the binding of MLL-AF4 to the *HOXA9* promoter. We also show that stimulation of leukemia cells with FGF2 increases nuclear level of FGFR2 in its phosphorylated form, as well as *HOXA9* and *MEIS1* expression. In contrast, preincubation with the ATP-mimetic inhibitor PD173074, before FGF2 stimulation, reduced FGFR2 nuclear amount and *HOXA9* and *MEIS1* transcript level, thereby indicating that MLL-AF4 aberrant activity depends on the nuclear availability of FGFR2. Overall, our study identifies FGFR2 as a new and promising therapeutic target in t(4;11) leukemia.

## 1. Introduction

The t(4;11) chromosomal translocation is a common cause of infant acute lymphoblastic leukemia (ALL) [1,2]. It fuses in-frame the *mixed-lineage leukemia* (*MLL,* aka *KMT2A*) and the *AF4/FMR2 Family Member 1* (*AFF1*) genes, located on chromosome 11 and 4 respectively, leading to fusion genes that encode chimeric oncoproteins—namely MLL-AF4 and the reciprocal AF4-MLL [1,2,3,4,5,6]. This aberration has been identified in utero and in neonatal blood, indicating it arises in the prenatal period; it is rare in adults (3–4%) and is correlated with a very poor prognosis [1,7]. Although 80% of patients exhibit both of the fusion proteins, only MLL-AF4 is essential for leukemic transformation and maintenance [8,9].

To exert its aberrant transcriptional activity, MLL-AF4 binds and deregulates the expression of key target genes involved in lymphocyte differentiation, including the homeobox A (*HOXA*) cluster genes and *MEIS1* [5,10,11,12]. Indeed, in many patients, survival of leukemic blasts depends on the maintenance of high expression levels of *HOXA9, HOXA8, HOXA7*, and *MEIS1* [12,13,14]; consistently, *HOXA9* silencing promotes apoptotic death in t(4;11) (q21;q23) lymphoblasts [15].

The AF4 protein, which is encoded by the *AFF1* gene, takes part in the AF4 family/ENL family/P-TEFb (AEP) protein complex that is crucial for chimera’s aberrant function. In addition to AF4, the AEP complex is formed by the transcriptional activators ENL, ELL, and AF5q—which are also common MLL fusion partners in human leukemia—as well as the positive elongation factor (P-TEFb) [16]. Through the direct interaction with the scaffold protein 14-3-3θ, AF4 and/or AF5q heterodimerize with MLL-AF4, thereby promoting the constitutive assembly of the AEP complex and the subsequent retrieval of RNA Pol II on target-gene promoters [17,18]. Moreover, MLL-AF4 forms a complex with DOT1L, a histone H3 lysine-79 (H3K79) methyltransferase, and other MLL fusion partners, such as AF9 and AF10 [11,18]. The recruitment of the AEP complex on target genes triggers the aberrant activity of DOT1L; in agreement, the epigenetic signature of H3K79me2, H3K27ac, and H3K4me3 marks all the MLL-AF4 target genes [11,18].

Consequently, the oncogenic potential of MLL-AF4 is mostly driven by the interaction with AF4 and its protein partners, which therefore represent promising therapeutic targets in t(4;11) leukemia. Interestingly, fibroblast growth factor receptor 2 (FGFR2) was found among the protein interactors of AF4 [19].

FGFR2 belongs to the family of receptor tyrosine kinases (RTKs), transmembrane-type receptors mainly localized on the cell surface with cytoplasmic tyrosine kinase domains [20]. It is able to recognize as ligands specific fibroblast growth factors (FGFs), with autocrine or paracrine action [21]. FGFs stimulate the intrinsic tyrosine kinase activity of the FGFRs and trigger various intracellular transduction signals that mediate multiple biological responses, including proliferation, differentiation, and cell survival [22].

The function of several RTKs is altered in different types of tumors and various drugs targeting the receptors and/or their downstream signaling pathways are already available and approved in clinical settings [23,24,25]. Of note, some RTK-related signaling pathways that influence cell growth and proliferation are activated in MLL-related leukemia [26,27,28].

Interestingly, various receptors, including RTKs and G-proteins coupled receptors (GPCRs), traffic from the cell surface to the nucleus [29,30,31,32,33]. In most cases, an intracellular domain fragment of the receptor translocates from the cell surface to the nucleus, whereas, for a few others, the intact receptor enters into the nucleus [28,29].

We herein analyze the function of nuclear FGFR2 in t(4;11) leukemia cells and its potential role as a molecular target for the treatment of this rare and poorly curable form of leukemia [7].

## 2. Results

We aimed to characterize the role of FGFR2 in t(4;11) leukemia. We analyzed the interaction between MLL-AF4 and FGFR2 and studied the effect of FGFR2 knockdown and inhibition on MLL-AF4 target gene expression.

### 2.1. FGFR2 Is a Nuclear Interactor of MLL-AF4

**Figure 1:**
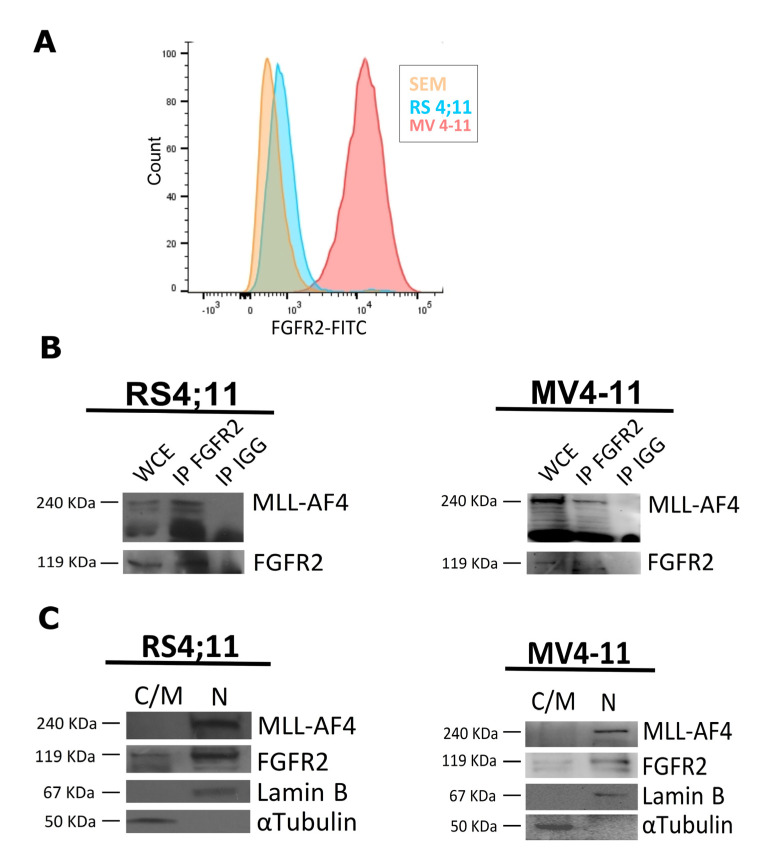
FGFR2 interacts with MLL-AF4 and localizes in the nucleus of t(4;11) leukemia cells. (**A**) Flow cytometry analysis carried out in different leukemia cell lines (SEM, RS4;11, and MV4-11) using anti-FGFR2 antibody and a fluorescein isothiocyanate (FITC)-conjugated secondary antibody. (**B**) Endogenous FGFR2 was immunoprecipitated from whole cellular extract (WCE) of RS4;11 and MV4-11 cells with anti-FGFR2 antibody (IP FGFR2) and with anti-IgG (IP IGG) antibodies, and isolated using A/G plus agarose beads; immunocomplexes were analyzed by western blot, with an anti-MLL antibody. (**C**) Twenty micrograms of cytosolic/membrane (C/M) and 50 µg of nuclear (N) proteins extracted from RS4;11 and MV4-11 cells were analyzed by western blot with anti-FGFR2 and anti-MLL antibodies; 〈–tubulin and lamin B were used as cytosolic and nuclear control proteins, respectively.

During a previous functional proteomic analysis performed in HEK293 cells, we found FGFR2 among the molecular partners of the AF4 protein [19]. Therefore, we wondered whether FGFR2 interacted also with the MLL-AF4 chimera in t(4;11) leukemia cell lines. Firstly, by flow cytofluorometry, we showed that FGFR2 was significantly represented on the cell surface of three t(4;11) leukemia cell lines that endogenously expressed MLL-AF4, namely RS4;11, SEM, and MV4-11 (Figure 1A). Therefore, we carried out coimmunoprecipitation experiments in RS4;11 and MV4-11 leukemia cells and found that endogenous FGFR2 interacted with endogenous MLL-AF4 (Figure 1B). 

In addition to being present on the cell surface, FGFR2 is known to have intracellular localization [34]. Thus, to better characterize the interaction between FGFR2 and MLL-AF4, we evaluated their cellular distribution. First, we analyzed cytosolic/membrane and nuclear protein fractions isolated from RS4;11 and MV4-11 cells by western blot. As expected, the MLL-AF4 oncoprotein was exclusively found in the nucleus, whereas FGFR2 was present in the cytosol/membrane—in agreement with its conventional function—and, significantly, also in the nuclear fraction (Figure 1C). These results indicate that FGFR2 and MLL-AF4 colocalize and therefore may interact within the nucleus of leukemia cells.

### 2.2. FGFR2 Silencing Affects Expression of MLL-AF4 Target Genes

**Figure 2:**
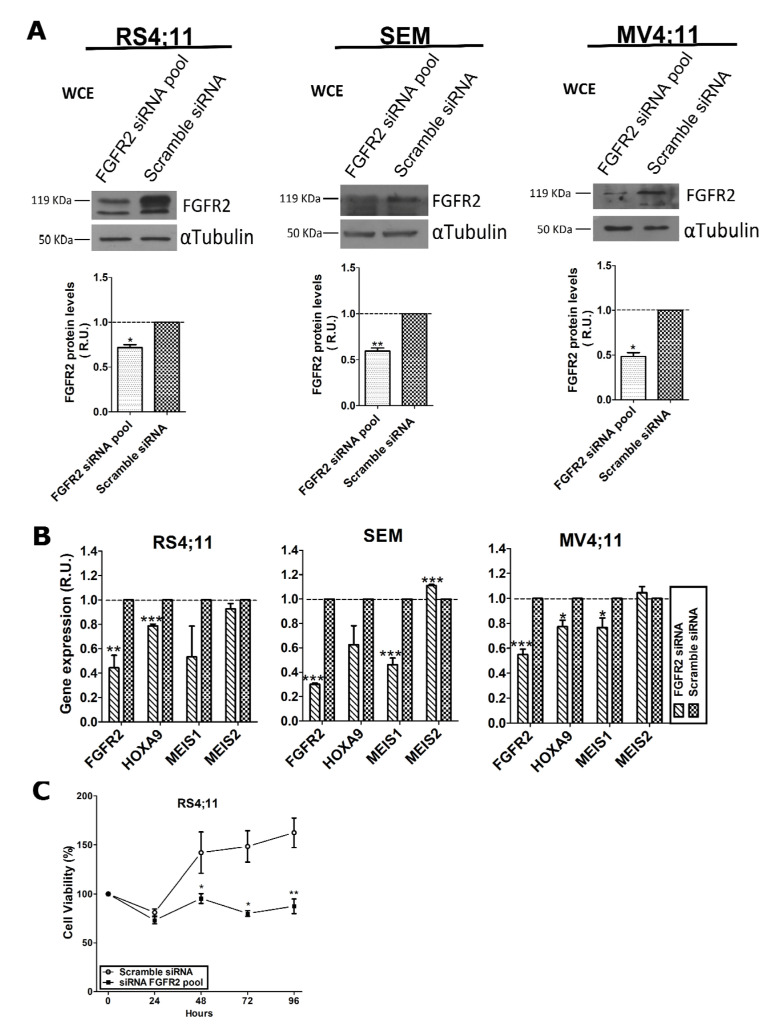
FGFR2 affects transcription of MLL-AF4 target genes in three t(4;11) cell lines. (**A**) Western blot analysis of total proteins (WCE) extracted 72 h after transfection with FGFR2-siRNA pool carried out with anti-FGFR2 antibody to evaluate residual FGFR2 amount; scramble siRNA represents the negative control; α-tubulin was used to normalize protein loading. (**B**) RT-qPCR analysis of *FGFR2*, *HOXA9*, *MEIS1*, and *MEIS2* transcript levels in FGFR2-silenced cells. Average values from at least three independent experiments are graphically reported as relative units (R.U.). Relative gene expression was normalized to *ACTB, POLR2A*, and *TUBA1A* genes and expression levels were determined using the 2^−ΔΔCt^ method. Statistical significance was calculated by one-way two-tail paired *t*-test. *p*-values are indicated as follows: * = *p* < 0.05; ** = *p* < 0.01; *** = *p* < 0.005. (**C**) Cell viability determined by MTT assay at 0, 24, 48, 72, and 96 h after transfection of cells with a FGFR2 siRNA pool and with scramble siRNA as a control. R.U., relative units.

Once FGFR2 was proven to be a protein partner of MLL-AF4, we wondered whether it contributed to MLL-AF4 transcriptional activity. To this aim, we first transfected RS4;11, SEM, and MV4-11 cell lines with a pool of specific small interfering RNA (siRNA) directed against the *FGFR2* transcript to knock down expression of the receptor. The same cell lines were also transfected with a scramble siRNA as a control. Seventy-two hours after transfection, total proteins were extracted and analyzed by western blot, which demonstrated a statistically significant reduction of FGFR2 in all cell lines (Figure 2A). 

Therefore, we extracted total RNA from silenced cells to analyze, by quantitative reverse transcription-polymerase chain reaction (RT-qPCR), transcript levels of *HOXA9* and *MEIS1*, two well-known MLL-AF4 target genes, and of *MEIS2*, which was not a chimera target gene. Results showed that *HOXA9* and *MEIS1*, but not *MEIS2*, expression was significantly reduced 72 h after FGFR2 silencing (Figure 2B). These results are in line with the assumption that MLL-AF4 transcriptional activity depends on FGFR2 availability.

To evaluate whether FGFR2 knockdown affected cell survival, we evaluated viability of RS4;11 cells, transfected with a FGFR2 siRNA pool and with scramble siRNA as a negative control, by MTT assay. Analysis was carried out 24, 48, 72, and 96 h after transfection and results were normalized to the untransfected cells (Figure 2C). Seventy-two hours after transfection, cell viability was significantly reduced in silenced cells and remained low until 96 h, whereas the viability of scrambled cells was comparable to the untransfected ones at 48, 72, and 96 h. This result strongly suggests that FGFR2 contributes to the survival of t(4;11) leukemia cells.

**Figure 3:**
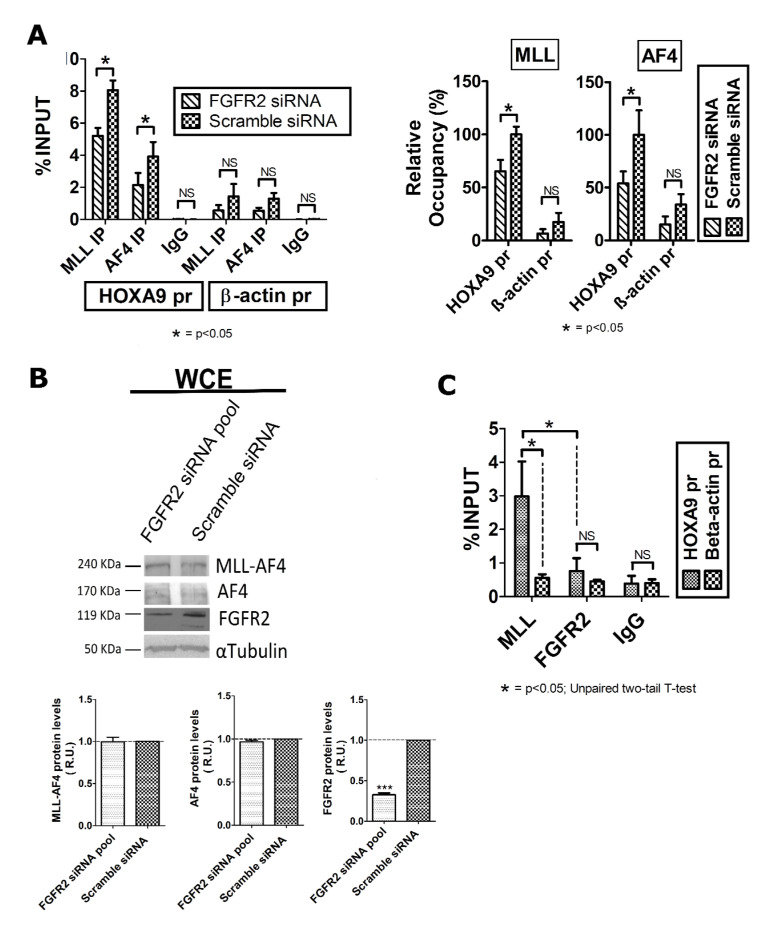
FGFR2 contributes to the interaction of AF4 and MLL-AF4 at the *HOXA9* promoter (HOXA9 pr), in RS4;11 leukemia cells. (**A**) ChIP assays performed to test the binding of AF4 and MLL-AF4 to *HOXA9* pr, after FGFR2 silencing. Scramble siRNA represents the negative control. (**B**) Western blot analysis of total protein (WCE) extracted 72 h after transfection of a FGFR2-siRNA pool carried out to reveal expression level of endogenous MLL-AF4, AF4, and FGFR2; scramble siRNA represents the negative control; α-tubulin was used to normalize protein loading. (**C**) Interaction of endogenous MLL and FGFR2 with the *HOXA9*pr. ChIP data are expressed as percentage of *HOXA9pr* and *β-actin* promoter (®-actin pr) in precipitated chromatin compared with the INPUT; IgG mix is the negative control. Results represent the average of three independent experiments. Error bars indicate the standard deviations. Statistical significance is calculated by one-way two tail paired *t*-test. *p*-values are indicated as follows: * = *p* < 0.05, *** *p* = < 0.005. NS, not significant. R.U., relative units.

It is widely proven that MLL-AF4 binds to the *HOXA9* promoter and activates the transcription of this gene in MLL-related leukemia [17,35]. Since MLL-AF4 is a protein interactor of FGFR2, and FGFR2 silencing decreased expression of *HOXA9,* we evaluated the binding efficiency of MLL-AF4 to the *HOXA9* promoter (*HOXA9*pr) in FGFR2-silenced RS4;11 cells. To this aim, we performed chromatin immunoprecipitation (ChIP) assays using an anti-MLL^N^ and an anti-AF4 antibody; a pool of nonspecific IgG served as a negative control (Figure 3A). More specifically, the anti-MLL^N^ antibody was directed against an N-terminal epitope of MLL wild type and therefore recognized also the MLL-AF4 oncoprotein; the anti-AF4 antibody served to reveal AF4, which was known to colocalize with MLL-AF4 on the *HOXA9* promoter [17]. Subsequent qPCR analysis revealed that the percentage of the *HOXA9*pr sequence in chromatin precipitated with both anti-AF4 and anti-MLL^N^ antibodies was significantly lower in FGFR2-silenced than in scrambled siRNA cells (Figure 3A).

Interestingly, western blot analysis performed on whole protein extracts from FGFR2-silenced cells showed that AF4 and MLL-AF4 protein levels were unaffected in comparison to the control cells (Figure 3B). Overall, these results demonstrate that silencing-induced FGFR2 deficiency, which has no effect on AF4 and MLL-AF4 total protein levels, weakens the binding of these two factors to the *HOXA9* promoter, thereby confirming that *HOXA9* expression specifically depends on the availability of FGFR2.

Subsequently, we investigated whether FGFR2 participated in the MLL-AF4 transcriptional machinery assembled on the *HOXA9* promoter. To this aim, we immunoprecipitated the chromatin in RS4;11 cells with an anti-FGFR2 antibody and looked for the *HOXA9pr* sequence by real time qPCR. Chromatin was also precipitated with the anti-MLL^N^ antibody and with a pool of nonspecific IgG, representing positive and negative control of ChIP specificity, respectively; a sequence within the *β-actin* gene promoter (*ACTB*pr) was amplified as a negative control. Data analysis revealed that the anti-FGFR2 antibody precipitated a percentage of *HOXA9pr* not significantly different from that of *ACTBpr.* In contrast, significant amount of DNA containing the *HOXA9pr* sequence was collected with the anti-MLL^N^ antibody (Figure 3C). These results indicate that despite FGFR2 and MLL-AF4 being protein interactors, they do not colocalize on chimera target gene promoters.

### 2.3. FGF2 Stimulates Nuclear Localization of FGFR2

**Figure 4:**
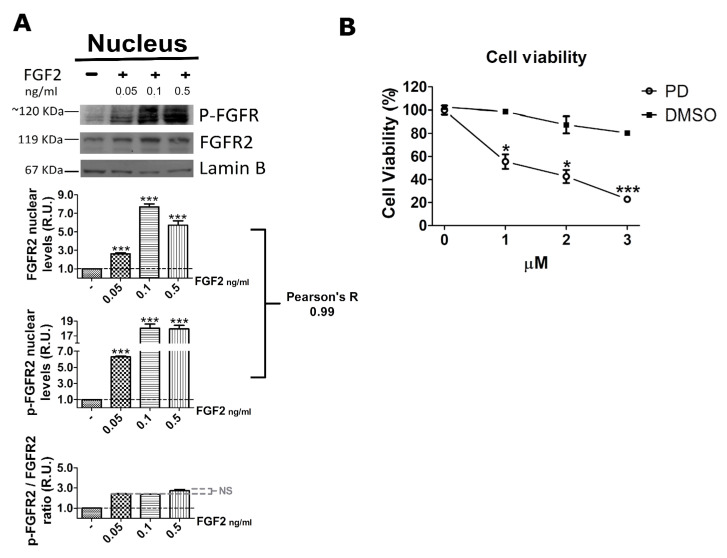
FGF2 promotes FGFR2 activation and nuclear translocation. (**A**) Western blot analysis of nuclear extracts obtained from serum-deprived RS4;11 cells stimulated with increasing concentrations of FGF2 performed with an anti-phospho-FGFR2 (p-FGFR2) or an anti-FGFR2 (FGFR2) antibody; lamin B was the control for protein loading; densitometric analysis of band intensity is graphically shown for FGFR2, p-FGFR2, and p-FGFR2/FGFR2 ratio. (**B**) Cell viability assay performed following treatment of RS4;11 cells with the FGFR inhibitor PD173074 (PD) and the vehicle (DMSO). Statistical significance was calculated by one-way two tail paired *t*-test. *p*-values are indicated as follows: * = *p* < 0.05, *** = *p* < 0.005. Pearson’s R = 0.99 is consistent with a positive correlation between the two set of values. NS, not significant. R.U., relative units.

To exert its conventional function, FGFR2 depends on cytoplasmic domain autophosphorylation consequent to the binding of its cognate agonists, i.e., fibroblast growth factors (FGFs) [34]. Moreover, several studies demonstrated that FGFR2 is also expressed in the nucleus [36] and that its ligand-dependent autophosphorylation triggers nuclear localization [37]. Therefore, we wondered whether FGFR2 nuclear localization relied on ligand-dependent autophosphorylation also in t(4;11) leukemia cells. To this aim, we first stimulated serum-starved RS4;11 cells with increasing concentrations of FGF2 and with the vehicle as a negative control, and then we purified nuclear proteins. Western blot analysis with anti-phospho-FGFR2 and anti-FGFR2 antibodies showed that FGF2 stimulation significantly increased both total and phosphorylated (p-FGFR2) levels of nuclear FGFR2 in a concentration-dependent manner, strongly suggesting that the nuclear increase in FGFR2 and pFGFR2 depends, at least in part, on the ligand concentration (Figure 4A). Notably, values of the p-FGFR2/FGFR2 ratio were very similar among the three tested concentrations of FGF2, indicating that the agonist-mediated activation of FGFR2 enhanced total and phosphorylated levels of the nuclear receptor proportionally. In fact, the Pearson’s R = 0.99 was consistent with a strong positive correlation between total and phosphorylated levels of the nuclear receptor (Figure 4A). Taken together, these results indicate that FGF2 stimulation triggers activation and nuclear translocation of FGFR2 in its phosphorylated form.

To further support the evidence that FGFR2 activation and nuclear translocation were ligand-dependent, we treated RS4;11 leukemia cells with PD173074, a synthetic compound belonging to the pyrido (2,3-d) pyrimidine class. PD173074 has a high selectivity for a set of RTKs, including FGFRs, and acts as an ATP mimetic; it replaces an ATP molecule in the binding pocket, thus blocking ligand-mediated autophosphorylation of FGFRs [38,39].

Preliminarily, we carried out a growth curve using increasing amount of PD173074 and, of interest, noted a dose-dependent impairment of leukemia cell viability, which therefore depended on RTK stimulation and autophosphorylation (Figure 4B).

**Figure 5:**
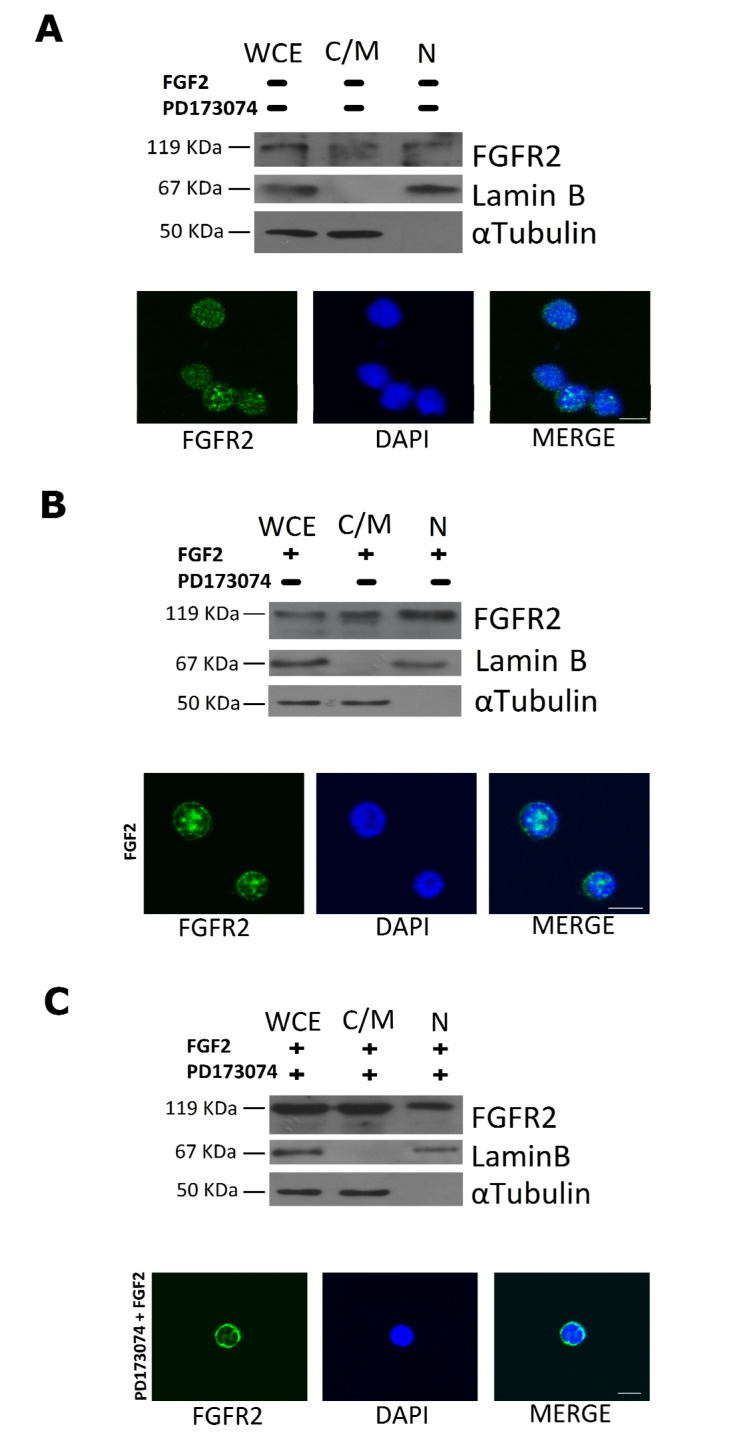
PD173074 inhibition affects cellular amount of FGFR2 in RS4;11 cells. RS4;11 cells were serum-starved for 12 h and preincubated for 1 h (**A**) with DMSO alone, (**B**) with DMSO and then stimulated with 0.1 ng/mL FGF2, or (**C**) with 2 µM of PD173074 and then stimulated with 0.1 ng/mL FGF2. Thirty micrograms of cytosolic/membrane proteins (C/M) and 50 µg of nuclear proteins (N) were analyzed by western blot with anti-FGFR2 antibody; αTubulin and Lamin B were used as loading control of cytosolic and nuclear proteins, respectively (upper panels); whole cellular extract (WCE). Immunofluorescence was performed with an anti-rabbit CY2 antibody to evaluate the localization of FGFR2, and DAPI, for nuclear staining (bottom panels). Scale bars: 10 μm.

Next, we incubated RS4;11 cells with the half inhibitory concentration of PD173074 for 1 h and then stimulated them with 0.1 ng/mL of FGF2 for 5 min. Western blot analysis performed on cytosolic/membrane and nuclear proteins showed that, differently from untreated cells (Figure 5A) and from cells preincubated with the vehicle and stimulated with FGF2 (Figure 5B), PD173074 treatment affected nuclear localization of FGFR2 (Figure 5C). These results were also confirmed by immunofluorescence assays (Figure 5A–C).

Taken together, the results in Figure 4 and Figure 5 clearly demonstrate that FGFR2 nuclear translocation depends on its ligand-dependent autophosphorylation, so that the measure of nuclear FGFR2 is also a measure of nuclear p-FGFR2.

**Figure 6:**
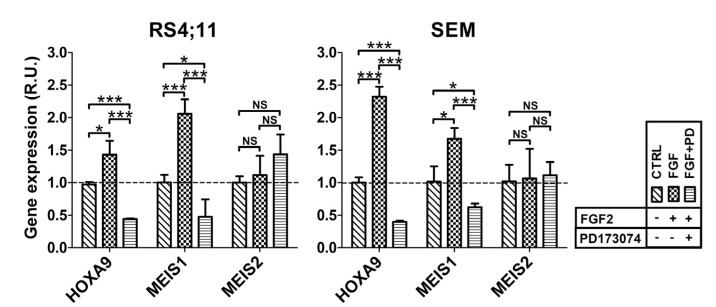
PD173074 inhibitor prevents FGF2-induced transcription of MLL-AF4 target genes. Graphs show transcript levels of MLL-AF4 target genes in leukemia cells, RS4;11 (left) and in SEM (right), stimulated with FGF2 (FGF) or pretreated with PD173074 (FGF + PD). Cells treated with DMSO (vehicle) are used as a control (CTRL); R.U., relative units; *p*-values are indicated as follows: * *p* < 0.05, *** *p* < 0.005.

Consistent with these findings and with previous evidence that the binding of MLL-AF4 to the *HOXA9* gene promoter depended on the nuclear availability of FGFR2 (Figure 2 and Figure 3), we expected that an FGF2-mediated nuclear increase in p-FGFR2 would affect the MLL-AF4 chimera transactivity. Therefore, we carried out RT-qPCR analysis to evaluate chimera target gene expression in RS4;11 and SEM stimulated with FGF2 or pretreated with the PD173074 inhibitor. As expected, FGF2 stimulation led to increased expression of *HOXA9* and *MEIS1 (*but not of *MEIS2*) and this overexpression was prevented when the cells were preincubated with PD173074 (Figure 6).

These data definitively demonstrate that FGF2-induced nuclear translocation of p-FGFR2 promotes aberrant activity of MLL-AF4.

## 3. Discussion

The t(4;11) chromosomal reciprocal translocation causes a very aggressive form of ALL, which is driven by the aberrant transcriptional activity of the MLL-AF4 chimera. We previously identified a few protein partners of AF4 and demonstrated that its direct interactor 14-3-3θ enhances the aberrant activity of MLL-AF4, thereby giving proof that AF4 interactors can affect chimera function [17,19]. Among the protein partners of AF4, we identified FGFR2 as being noteworthy [19].

Significant amount of this receptor is expressed on the plasma membrane of three different t(4;11) leukemia cell lines, i.e., RS4;11, MV4-11, and SEM (Figure 1A). FGFRs are involved in multiple myeloma, in myeloproliferative disorder, and, importantly, in B-cell precursor (BCP) ALL, the latter including the MLL-rearranged leukemias, thereby supporting their crucial role in hematologic malignancies [40,41,42]. Interestingly, FGFR signaling can contribute to prednisolone resistance in BCP ALL cells; however, as activating mutations in this RTK family are very rare, information concerning their role in ALL is limited [41].

Herein, we showed that endogenous FGFR2 coimmunoprecipitates endogenous MLL-AF4 in t(4;11) leukemia cells, thereby demonstrating that, similarly to AF4, it participates in the MLL-AF4 protein complex [14,16,17]. Since MLL-AF4 and AF4 exert their activity in the nucleus, binding with FGFR2 is functional only if the receptor has a nuclear localization.

Similarly to other plasma membrane receptors, FGFRs have also been found in the nucleus [29,31,32,33,36,37]. Indeed, after its activation by extracellular ligands, FGFR1 moves to the nucleus where it regulates gene transcription in cooperation with cyclic AMP-responsive element binding protein by increasing activity of Pol II and histone acetylation [43]. Moreover, in human breast cancer tissues, FGF stimulation causes nuclear translocation of FGFR2, which interacts with the transcriptional factor STAT5 and increases expression of STAT5 target genes and proteins [33].

Besides confirming that MLL-AF4 is exclusively present in the nucleus, we revealed that an appreciable amount FGFR2 consistently localizes in the nuclear compartment of t(4;11) leukemia cells, as evidenced by western blot and immunofluorescence analyses (Figure 1C and Figure 5A). Therefore, the interaction between the two proteins can actually take place in the nucleus.

The interaction between FGFR2 and MLL-AF4 chimera is particularly interesting, especially based on the evidence that FGFR2 silencing reduces transcript levels of the chimera target genes, *HOXA9* and *MEIS1*, in t(4;11) leukemia cells (Figure 2). In agreement with the previous evidence that reduced expression of *HOXA9* and *MEIS1* impairs viability of leukemia cells [15,44], FGFR2 knockdown negatively affected the proliferative rate of RS4;11 leukemia cells (Figure 2C). Obviously, due to its main role in cellular growth, we cannot attribute this effect entirely to the probable reduced interaction between FGFR2 and MLL-AF4. However, our ChIP experiments demonstrated that FGFR2 deficiency reduced the binding of AF4 and of MLL-AF4 to the *HOXA9* promoter (Figure 3A). Nevertheless, further ChIP experiments performed with the anti-FGFR2 antibody did not detect FGFR2 on the *HOXA9* gene promoter (Figure 3B). Based on this evidence, we conclude that—despite FGFR2 not localizing directly on the chromatin—nuclear availability of the receptor is crucial for the binding of AF4 and MLL-AF4 to the *HOXA9* promoter, which in turn is necessary for the chimera target gene expression.

As our preliminary data suggested that reducing FGFR2 nuclear import is potentially therapeutic for t(4;11) leukemia, it is relevant to understand how FGFR2 enters into the nucleus. In human breast cancer, FGFR2 moves to the nucleus after activation by extracellular ligands; however, the commercial ATP pocket inhibitor PD173074 blocks this agonist-induced FGFR2 nuclear translocation and inhibits RTK activity and downstream pathways [30,36]. In general, PD173074 and similar drugs are able to inhibit FGF signaling in vivo [39,40].

We show that stimulation with FGF2, which is highly expressed in the hematopoietic and stromal compartments of the bone marrow [31,41], triggered phosphorylation and nuclear translocation of FGFR2 in t(4;11) leukemia cells (Figure 4B). On the other hand, when activation of FGFR2 was blocked with PD173074, a smaller amount of FGFR2 was present in the nucleus of t(4;11) leukemia cells with respect to the untreated ones, as shown by our western blot and immunofluorescence analyses (Figure 5). In agreement and of further consequence, stimulation of t(4;11) cells with FGF2 led to increased transcription of the MLL-AF4 target genes, *HOXA9* and *MEIS1*, which was prevented by pretreatment of the cells with the PD173074 inhibitor (Figure 6). Consistently, PD173074 treatment also impaired viability of the leukemia cells (Figure 4B).

**Figure 7:**
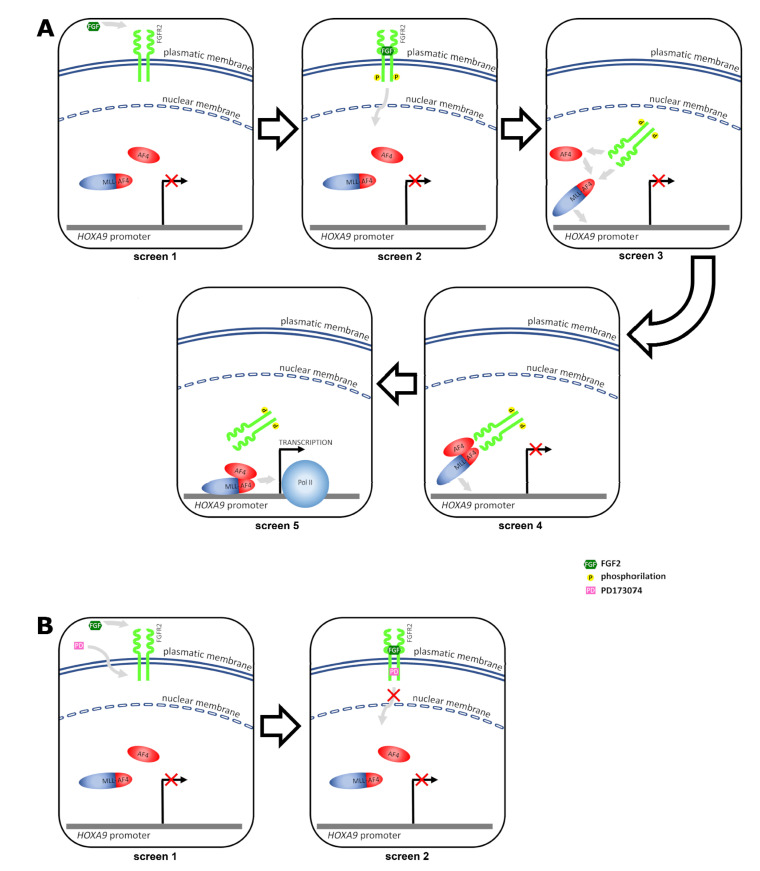
Nuclear cross-talk between FGFR2 and MLL-AF4 promotes aberrant transcription of *HOXA9* in leukemia cells. (**A**) screen 1: in basal condition, *HOXA9* transcription is not stimulated; screen 2: FGF2 binds FGFR2 and triggers its phosphorylation; screen 3: p-FGFR2 enters the nucleus and interacts with both the MLL-AF4 chimera and AF4; screen 4: p-FGFR2 promotes the binding of the MLL-AF4/AF4 complex to the *HOXA9* promoter; screen 5: the MLL-AF4/AF4 complex activates RNA Pol II-dependent transcription of *HOXA9*. (**B**) screen 1: the PD173074 inhibitor enters the cell, binds the ATP pocket of FGFR2, and prevents FGF2-mediated autophosphorylation of the receptor; screen 2: unphosphorylated FGFR2 does not enter the nucleus and therefore cannot promote the MLL-AF4-driven transcription of *HOXA9*.

Based on our overall results, we propose a model illustrating how nuclear cross-talk between FGFR2 and MLL-AF4 promotes aberrant transcription of MLL-AF4 target genes in leukemia cells (Figure 7).

In our model, the ligand FGF2 triggers phosphorylation and nuclear entry of FGFR2. In the nucleus, the activated receptor interacts with MLL-AF4 and AF4 and promotes the binding of the MLL-AF4/AF4 complex to the target gene promoter. Lastly, the MLL-AF4/AF4 complex recruits RNA Pol II on chromatin and activates gene transcription (Figure 7A). Treatment of leukemia cells with the PD173074 inhibitor, which binds the ATP pocket of FGFR2, prevents FGF2-mediated nuclear entry of pFGFR2 and, consequently, also transcriptional activation of MLL-AF4 target genes (Figure 7B). Similarly, specific silencing of FGFR2 leads to impaired chimera target gene transcription.

In conclusion, our study adds to the growing body of evidences that MLL-AF4, the oncogenic chimera typical of the t(4;11) ALL, promotes its aberrant transcriptional activity through the recruitment of nuclear molecular partners that, like FGFR2, consequently acquire an opportunistic oncogenic function. As our overall data give proof that the phosphorylation-dependent nuclear translocation of FGFR2 plays a key role in the leukemogenic mechanism, the use of FGFR-specific inhibitors or of molecules specifically able to block the nuclear import of the receptor, as well as the interaction between FGFR2 and the MLL-AF4 chimera, may be promising avenues to design new therapeutic strategies. Therefore, we realistically consider FGFR2 a novel and useful target for treatment of this very aggressive form of hematopoietic malignancy.

## 4. Materials and Methods

### 4.1. Antibodies

The following antibodies were used: mouse monoclonal anti-mouse α tubulin; rabbit polyclonal anti-FGFR2; goat polyclonal anti-lamin B (Santa Cruz Biotechnology, Inc., Dallas, TX, USA); mouse monoclonal anti-MLL (Upstate Biotechnology, Inc., New York, NY, USA); human phospho-FGFR1-4 (Y653/Y654) antibody (R&D Systems, Minneapolis, MN, USA); horseradish peroxidase-conjugated anti-mouse, anti-rabbit, and anti-goat IgG secondary antibodies (GE Healthcare Italia, Milan, Italy); anti-rabbit IgG, Cy2-conjugated (Merck KGaA, Darmstadt, Germany), and anti-rabbit IgG FITC-conjugated (Thermo Fisher Scientific, Waltham, MA, USA).

### 4.2. Cell Lines

Leukemia cell lines were obtained from the Cell Culture Facility of CEINGE - Advanced Biotechnologies (Naples, Italy). RS4;11, SEM, and MV4-11 harbor the t(4;11) chromosomal rearrangement and express endogenous MLL-AF4 chimera. The RS4;11 acute lymphoblastic leukemia cells were grown at 37 °C, 5% CO_2_, in minimum essential medium (MEM) (Sigma-Aldrich, St. Louis, MO, USA) supplemented with 10% FBS and 10 mL/L penicillin/streptomycin (Sigma-Aldrich, St. Louis, MO, USA); SEM acute lymphoblastic leukemia cells were grown at 37 °C, 5% CO_2_, in Iscove’s MDM (Sigma-Aldrich) supplemented with 10% FBS and 10 mL/L penicillin/streptomycin; MV4-11 acute monocytic leukemia cells and 697 acute lymphoblastic leukemia cells were grown in RPMI (Sigma-Aldrich) supplemented with 20% FBS and 10 mL/L penicillin/streptomycin.

### 4.3. Flow Cytometry

Leukemia cells (1 × 10^6^) were incubated with anti-FGFR2 antibody for 1 h. After several washes with PBS, cells were treated with fluorescein isothiocyanate (FITC)-secondary antibody for 30 min and read using a BD FACSCanto II (BD Biosciences-US, San Jose, CA, USA) flow cytometer.

### 4.4. Protein Extraction, Subcellular Fraction Isolation, Immunoprecipitation, and Western Blot Analysis

For immunoprecipitation (IP) experiments, RS4;11 and MV4-11 cells were lysed in IP buffer, as previously described [21], and protein extract was incubated overnight at 4 °C with anti-FGFR2 antibody (2 µg per 5 mg of total proteins). Subsequently, the protein mixture was incubated with 30 mL of protein A/G PLUS-Agarose (Santa Cruz Biotechnology, Inc., Dallas, TX, USA) for each microgram of antibody [17].

Fractioned protein extracts containing cytosolic/membrane and nuclear proteins were obtained using a Qproteome Nuclear Protein Kit (Qiagen Italia, Milan, Italy) as elsewhere described [45,46]. Briefly, cells were washed twice with ice-cold PBS, detached by scraping, and centrifuged for 5 min at 450 RCF. They were then lysed by incubation for 15 min in hypotonic nuclear lysis (NL) buffer, supplemented with protease inhibitor solution and 0.1 M DTT. Detergent solution was then added and, after brief shaking, the cell suspension was centrifuged for 5 min at 10,000 RCF. The supernatant, containing the cytosol/membrane proteins, was stored and used for further analyses. The cell nuclei, contained in the pellet, were washed by resuspension in the NL buffer and subsequent centrifugation for 5 min at 10,000 RCF. The nuclear pellet was resuspended in the buffer NX1, supplemented with protease inhibitor solution, and incubated for 30 min under shaking. The suspension was then centrifuged for 10 min at 12,000 RCF and the supernatant, containing the nuclear proteins, was stored and used for further analyses. Buffers, solutions, and reagents were provided by the Qproteome Nuclear Protein Kit (Qiagen Italia, Milan, Italy) and all the procedure steps were carried out at 4 °C. Cytosolic/membrane and nuclear proteins were analyzed with proper antibodies to verify the quality of the fractionation procedure.

Either 40 µg of WCE, or 50 µg of nuclear extract, or 20–30 µg of cytosolic/membrane extract, or 15 µL of sample from IP experiments were loaded onto SDS/PAGE. After electrophoretic separation, proteins were transferred onto nitrocellulose membrane and analyzed with appropriate primary antibodies. Protein signals were visualized with the ECL Plus detection system (GE Healthcare Italia, Milan, Italy) and protein signal intensities were quantified with the ImageJ 1.46 software. Average values from at least three independent experiments were graphically reported as relative units (R.U.).

### 4.5. Small Interfering RNA (SiRNA)

RS4;11, SEM, and MV4-11 leukemia cells were transfected with 100 nM FGFR2-specific siRNAs ON-TARGET plus SMARTpool (Qiagen Italia, Milan, Italy) by electroporation (Bio-Rad Laboratories S.r.l., Milan, Italy) in MEM Eagle (Sigma-Aldrich, St. Louis, MO, USA). All Stars Negative Control siRNA scramble (Qiagen) was the nonsense control. After incubation for 15 min at room temperature, cells were cultured under standard conditions and harvested after 72 h to isolate total RNA and total proteins [17].

### 4.6. Total RNA Isolation, Reverse Transcription (RT), and Real Time Polymerase Chain Reaction (PCR) 

Total RNA was extracted from silenced cells (RS4;11, SEM, and MV4-11) with the Nucleo Spin RNA II kit (Macherey–Nagel GmbH & Co. KG, Dueren, Germany); 200 ng of RNA were reverse transcribed using SuperScript III and random-hexamers oligo-dT (Thermo Fisher Scientific, Waltham, MA, USA). Real time PCR was carried out in an iCycler iQ Real Time PCR Thermal Cycler (Bio-Rad Laboratories, Hercules, CA, USA) using SYBR Green Master Mix (Bio-Rad Laboratories, Hercules, CA, USA) and specific primer pairs. To measure expression level of *HOXA9*, *MEIS1*, and *MEIS2*, the primer pairs used were described previously [17]; for the dosage of FGFR2 the following primers were used: FGFR2-F: 5′-GTCAGCTGGGGTCGTTTC-3′; FGFR2-R: 5′-TCATGTTTTAACACTGCCGTT-3′. Gene expression was normalized to *POLR2A*, *ACTB*, and *TUBA1A* genes and determined using the 2^−ΔΔCt^ method. Average values from at least three independent experiments were graphically reported as relative units (R.U.).

### 4.7. Cell Viability Assays

RS4;11 cells (4 × 10^4^ per well) were transfected with FGFR2-specific siRNAs and siRNA nonsense control, and seeded in a 96-well plate. Cell viability was assessed by the 3-(4,5-dimethyl-2-thiazolyl)-2,5-diphenyl-2H-tetrazolium bromide (MTT) (Sigma Aldrich) method [17]. MTT (0.5 mg/mL of fresh media) was added to the cells (RS4;11) 24, 48, 72, and 96 h after transfection. Absorbance was read at 570 nm using a Spectramax spectrophotometer (Molecular Devices, San Jose, CA, USA).

To calculate the half-maximal inhibitory concentration (IC_50_) of the PD173074, 2 million RS4;11 cells were plated in a 96-multiwell plate in serum-free medium and treated with various concentrations of the inhibitor. Then, 0.1 ng/mL of FGF2 was added to the medium and 72 h after treatment MTT was added (0.5 mg/mL of fresh media). Absorbance was read at 570 nm.

### 4.8. Chromatin Immunoprecipitation (ChIP) Assay

RS4;11 cells (30 × 10^6^) were fixed in MEM medium containing 1% formaldehyde for 10 min at room temperature; the reaction was stopped by glycine quenching (125 mM final concentration). Nuclei were collected, digested in 50 mM Tris-HCL pH 8.1, 10 mM EDTA, 10% SDS, and then sonicated (3 cycles, consisting of 30 s with and without sonication) using a Microson XL ultrasonic cell disruptor (Misonix Inc., Farmingdale, NY, USA). Proteins tied to DNA fragments (ranging from 100–600 bp) were pulled-down overnight at 4 °C using appropriate antibodies, then mixed with protein-G magnetic beads (Santa Cruz Biotechnology Inc., Dallas, TX, USA) and incubated for 2 h. Beads were washed with ChIP buffer (10 mM Tris-HCl pH 8.1, 1 mM EDTA, 10% SDS, 0.5% EGTA, 140 mM NaCl, 10× Na-deoxycholate, 100× Triton). Immunoprecipitates were dissolved in elution buffer (0.5 M EDTA, 1 M Tris-HCl pH 8.0) and DNA was isolated by phenol/chloroform/isoamyl alcohol extraction and ethanol precipitation. Real time quantitative PCR (RT-qPCR) was performed with 1 µL of DNA using a custom-made primer set [17].

### 4.9. Treatment of t(4;11) Leukemia Cells with FGF2 and PD173074 Inhibitor

One million RS4;11 cells were plated in serum-free medium for 12 h and then stimulated with various concentrations of FGF2 (0.05–0.1–0.5 ng/mL). 50 µg of nuclear protein were loaded onto 10% SDS-PAGE for the western blot, performed with an anti-phospho-FGFR2 (P-FGFR2) or an anti-FGFR2 (FGFR2) antibody [47]. One million RS4;11 cells were plated in serum-free medium for 12 h, treated with 2 µM PD173074 (Sigma Aldrich) for 1 h, and then stimulated with 0.1 ng/mL of FGF2 (Sigma-Aldrich). Cells were harvested 72 h after treatment and total, cytosolic/membrane, and nuclear proteins were extracted [46,47].

### 4.10. Immunofluorescence Analysis

RS4;11 cells, treated with 2 µM PD173074 and stimulated with 0.1 ng/mL FGF2, as described in the previous paragraph, were fixed in 2% paraformaldehyde (Sigma Aldrich, St. Louis, MO, USA), permeabilized with 1% BSA in PBS, incubated with the anti-FGFR2 antibody, and subsequently treated with a CY2-conjugated secondary antibody. DAPI solution (Thermo Fisher Scientific) was used for nuclear staining. Cells were mounted on a slide and analyzed by confocal microscopy (LSM 510, Zeiss, München, Germany).

### 4.11. Statistical Analysis

All the data presented are expressed as mean ± standard error mean (SEM) and are representative of three or more independent experiments. The data of repeated experiments were analyzed using one-way Student‘s *t*-test (for independent samples). 

## Data Availability

Not Applicable.

## References

[1] Daser A., Rabbitts T.H. (2005). The versatile mixed lineage leukaemia gene *MLL* and its many associations in leukaemogenesis. Semin. Cancer Biol..

[2] Meyer C., Hofmann J., Burmeister T., Gröger D., Park T.S., Emerenciano M., Pombo de Oliveira M., Renneville A., Villarese P., Macintyre E. (2013). The MLL recombinome of acute leukemias in 2013. Leukemia.

[3] Bursen A., Schwabe K., Rüster B., Henschler R., Ruthardt M., Dingermann T., Marschalek R. (2010). The AF4.MLL fusion protein is capable of inducing ALL in mice without requirement of MLL.AF4. Blood.

[4] Prieto C., Marschalek R., Kühn A., Bursen A., Bueno C., Menéndez P. (2017). The AF4-MLL fusion transiently augments multilineage hematopoietic engraftment but is not sufficient to initiate leukemia in cord blood CD34^+^ cells. Oncotarget.

[5] Wilkinson A.C., Ballabio E., Geng H., North P., Tapia M., Kerry J., Biswas D., Roeder R.G., Allis C.D., Melnick A. (2013). RUNX1 Is a key target in t(4;11) leukemias that contributes to gene activation through an AF4-MLL complex interaction. Cell Rep..

[6] Bueno C., Calero-Nieto F.J., Wang X., Valdés-Mas R., Gutiérrez-Agüera F., Roca-Ho H., Ayllon V., Real P.J., Arambile D., Espinosa L. (2019). Enhanced hemato-endothelial specification during human embryonic differentiation through developmental cooperation between AF4-MLL and MLL-AF4 fusions. Haematologica.

[7] Britten O., Ragusa D., Tosi S., Kamel Y.M. (2019). MLL-Rearranged Acute Leukemia with t(4;11)(q21;q23)—Current Treatment Options. Is There a Role for CAR-T Cell Therapy?. Cells.

[8] Kowarz E., Burmeister T., Lo Nigro L., Jansen M.W., Delabesse E., Klingebiel T., Dingermann T., Meyer C., Marschalek R. (2007). Complex MLL rearrangements in t(4;11) leukemia patients with absent AF4.MLL fusion allele. Leukemia.

[9] Thomas M., Gessner A., Vornlocher H.P., Hadwiger P., Greil J., Heidenreich O. (2005). Targeting MLL-AF4 with short interfering RNAs inhibits clonogenicity and engraftment of t(4;11)-positive human leukemic cells. Blood.

[10] Marschalek R. (2011). Mechanisms of leukemogenesis by MLL fusion proteins. Br. J. Haematol..

[11] Godfrey L., Kerry J., Thorne R., Repapi E., Davies J.O., Tapia M., Ballabio E., Hughes J.R., Geng H., Konopleva M. (2016). MLL-AF4 binds directly to a BCL-2 specific enhancer and modulates H3K27 acetylation. Exp. Hematol..

[12] Collins C.T., Hess J.L. (2016). Deregulation of the HOXA9/MEIS1 axis in acute leukemia. Curr. Opin. Hematol..

[13] Stam R.W., Schneider P., Hagelstein J.A.P., van der Linden M.H., Stumpel D.J.P.M., de Menezes R.X., de Lorenzo P., Valsecchi M.G., Pieters R. (2010). Gene expression profiling-based dissection of MLL translocated and MLL germline acute lymphoblastic leukemia in infants. Blood.

[14] Yokoyama A. (2016). Transcriptional activation by MLL fusion proteins in leukemogenesis. Exp. Hematol..

[15] Orlovsky K., Kalinkovich A., Rozovskaia T., Shezen E., Itkin T., Alder H., Ozer H.G., Carramusa L., Avigdor A., Volinia S. (2011). Down-regulation of homeobox genes MEIS1 and HOXA in MLL-rearranged acute leukemia impairs engraftment and reduces proliferation. Proc. Natl. Acad. Sci. USA.

[16] Yokoyama A., Lin M., Naresh A., Kitabayashi I., Cleary M.L. (2010). A higher-order complex containing AF4 and ENL family proteins with P-TEFb facilitates oncogenic and physiologic MLL-dependent transcription. Cancer Cell.

[17] Fioretti T., Cevenini A., Zanobio M., Raia M., Sarnataro D., Salvatore F., Esposito G. (2019). Crosstalk between 14-3-3θ and AF4 enhances MLL-AF4 activity and promotes leukemia cell proliferation. Cell. Oncol..

[18] Okuda H., Stanojevic B., Kanai A., Kawamura T., Takahashi S., Matsui H., Takaori-Kondo A., Yokoyama A. (2017). Cooperative gene activation by AF4 and DOT1L drives MLL-rearranged leukemia. J. Clin. Investig..

[19] Esposito G., Cevenini A., Cuomo A., de Falco F., Sabbatino D., Pane F., Ruoppolo M., Salvatore F. (2011). Protein network study of human AF4 reveals its central role in RNA Pol II-mediated transcription and in phosphorylation-dependent regulatory mechanisms. Biochem. J..

[20] Lemmon M.A., Schlessinger J. (2010). Cell signaling by receptor tyrosine kinases. Cell.

[21] Lonic A., Barry E., Quach C., Kobe B., Saunders N., Guthridge M.A. (2008). Fibroblast Growth Factor Receptor 2 Phosphorylation on Serine 779 Couples to 14-3-3 and Regulates Cell Survival and Proliferation. Mol. Cell. Biol..

[22] Xie Y., Su N., Yang J., Tan Q., Huang S., Jin M., Ni Z., Zhang B., Zhang D., Luo F. (2020). FGF/FGFR signaling in health and disease. Signal Transduct. Target. Ther..

[23] Montor W.R., Salas A.R.O.S.E., Melo F.H.M. (2018). Receptor tyrosine kinases and downstream pathways as druggable targets for cancer treatment: The current arsenal of inhibitors. Mol. Cancer.

[24] Yamaoka T., Kusumoto S., Ando K., Ohba M., Ohmori T. (2018). Receptor Tyrosine Kinase-Targeted Cancer Therapy. Int. J. Mol. Sci..

[25] Korc M., Friesel R.E. (2009). The role of fibroblast growth factors in tumor growth. Curr. Cancer Drug Targets.

[26] Esposito M.T. (2019). The Impact of PI3-kinase/RAS Pathway Cooperating Mutations in the Evolution of KMT2A-rearranged Leukemia. HemaSphere.

[27] Whelan J.T., Ludwig D.L., Bertrand F.E. (2008). HoxA9 induces insulin-like growth factor-1 receptor expression in B-lineage acute lymphoblastic leukemia. Leukemia.

[28] Nakanishi H., Nakamura T., Canaani E., Croce C.M. (2007). ALL1 fusion proteins induce deregulation of EphA7 and ERK phosphorylation in human acute leukemias. Proc. Natl. Acad. Sci. USA.

[29] Stachowiak M.K., Maher P.A., Stachowiak E.K. (2007). Integrative nuclear signaling in cell development—A role for FGF receptor-1. DNA Cell Biol..

[30] Cerliani J.P., Guillardoy T., Giulianelli S., Vaque J.P., Gutkind J.S., Vanzulli S.I., Martins R., Zeitlin E., Lamb C.A., Lanari C. (2011). Interaction between FGFR-2, STAT5, and progesterone receptors in breast cancer. Cancer Res..

[31] Tuzon C.T., Rigueur D., Merrill A.E. (2019). Nuclear Fibroblast Growth Factor Receptor Signaling in Skeletal Development and Disease. Curr. Osteoporos. Rep..

[32] Cattaneo F., Parisi M., Fioretti T., Sarnataro D., Esposito G., Ammendola R. (2016). Nuclear localization of Formyl-Peptide Receptor 2 in human cancer cells. Arch. Biochem. Biophys..

[33] Carpenter G., Liao H.J. (2013). Receptor tyrosine kinases in the nucleus. Cold Spring Harb. Perspect. Biol..

[34] Porębska N., Latko M., Kucińska M., Zakrzewska M., Otlewski J., Opaliński Ł. (2018). Targeting cellular trafficking of fibroblast growth factor receptors as a strategy for selective cancer treatment. J. Clin. Med..

[35] Kerry J., Godfrey L., Repapi E., Tapia M., Blackledge N.P., Ma H., Ballabio E., O’Byrne S., Ponthan F., Heidenreich O. (2017). MLL-AF4 spreading identifies binding sites that are distinct from super-enhancers and that govern sensitivity to DOT1L inhibition in leukemia. Cell Rep..

[36] Martin A.J., Grant A., Ashfield A.M., Palmer C.N., Baker L., Quinlan P.R., Purdie C.A., Thompson A.M., Jordan L.B., Berg J.B. (2011). FGFR2 protein expression in breast cancer: Nuclear localisation and correlation with patient genotype. BMC Res. Notes.

[37] Schmahl J., Kim Y., Colvin J.S., Ornitz D.M., Capel B. (2004). Fgf9 induces proliferation and nuclear localization of FGFR2 in Sertoli precursors during male sex determination. Development.

[38] Bansal R., Magge S., Winkler S. (2003). Specific inhibitor of FGF receptor signaling: FGF-2-mediated effects on proliferation, differentiation, and MAPK activation are inhibited by PD173074 in oligodendrocyte-lineage cells. J. Neurosci. Res..

[39] Pardo O.E., Latigo J., Jeffery R.E., Nye E., Poulsom R., Spencer-Dene B., Lemoine N.R., Stamp G.W., Aboagye E.O., Seckl M.J. (2009). The fibroblast growth factor receptor inhibitor PD173074 blocks small cell lung cancer growth in vitro and in vivo. Cancer Res..

[40] Chae Y.K., Ranganath K., Hammerman P.S., Vaklavas C., Mohindra N., Kalyan A., Matsangou M., Costa R., Carneiro B., Villaflor V.M. (2017). Inhibition of the fibroblast growth factor receptor (FGFR) pathway: The current landscape and barriers to clinical application. Oncotarget.

[41] Jerchel I.S., Hoogkamer A.Q., Ariës I.M., Boer J.M., Besselink N.J.M., Koudijs M.J., Pieters R., den Boer M.L. (2019). Fibroblast growth factor receptor signaling in pediatric B-cell precursor acute lymphoblastic leukemia. Sci. Rep..

[42] Kasbekar M., Nardi V., Dal Cin P., Brunner A.M., Burke M., Chen Y.B., Connolly C., Fathi A.T., Foster J., Macrae M. (2020). Targeted FGFR inhibition results in a durable remission in an FGFR1-driven myeloid neoplasm with eosinophilia. Blood Adv..

[43] Stachowiak M.K., Birkaya B., Aletta J.M., Narla S.T., Benson C.A., Decker B., Stachowiak E.K. (2015). Nuclear FGF receptor-1 and CREB binding protein: An integrative signaling module. J. Cell. Physiol..

[44] Faber J., Krivtsov A.V., Stubbs M.C., Wright R., Davis T.N., van den Heuvel-Eibrink M., Zwaan C.M., Kung A.L., Armstrong S.A. (2009). HOXA9 is required for survival in human MLL-rearranged acute leukemias. Blood.

[45] Cattaneo F., Castaldo M., Parisi M., Faraonio R., Esposito G., Ammendola R. (2018). Formyl Peptide Receptor 1 Modulates Endothelial Cell Functions by NADPH Oxidase-Dependent VEGFR2 Transactivation. Oxid. Med. Cell. Longev..

[46] Castaldo M., Zollo C., Esposito G., Ammendola R., Cattaneo F. (2019). NOX2-Dependent Reactive Oxygen Species Regulate Formyl-Peptide Receptor 1-Mediated TrkA Transactivation in SH-SY5Y Cells. Oxid. Med. Cell. Longev..

[47] Cattaneo F., Russo R., Castaldo M., Chambery A., Zollo C., Esposito G., Pedone P.V., Ammendola R. (2019). Phosphoproteomic analysis sheds light on intracellular signaling cascades triggered by Formyl-Peptide Receptor 2. Sci. Rep..

